# Creation and Use of Highly Adaptive Productive and Technological Red Currant Genotypes to Improve the Assortment and Introduction into Different Ecological and Geographical Zones

**DOI:** 10.3390/plants11060802

**Published:** 2022-03-17

**Authors:** Olga Panfilova, Ibrahim Kahramanoğlu, Gabrijel Ondrasek, Volkan Okatan, Nelly Ryago, Mikhail Tsoy, Olga Golyaeva, Sergey Knyazev

**Affiliations:** 1 Russian Research Institute of Fruit Crop Breeding (VNIISPK), Zhilina 302530, Russia; ryago@vniispk.ru (N.R.); nauka@vniispk.ru (M.T.); golyaeva@vniispk.ru (O.G.); info@vniispk.ru (S.K.); 2 Department of Horticulture, Faculty of Agricultural Sciences and Technologies, European University of Lefke, Gemikonagi, Northern Cyprus, Mersin 99780, Turkey; ibrahimcy84@yahoo.com; 3 Faculty of Agriculture, University of Zagreb, 10000 Zagreb, Croatia; gondrasek@agr.hr; 4 Department of Horticulture, Faculty of Agriculture, Eskişehir Osmangazi University, Eskişehir 26160, Turkey; okatan.volkan@gmail.com

**Keywords:** genetic collection, adaptation, heat resistance, winter hardiness, water regime, productivity, stability, diseases and pests, cultivar

## Abstract

Global climate change with the cyclicity of natural and climatic processes in the growing season of berry plants, causes weakening at the defense system to (a)biotic stressors, which actualize the need for accelerated cultivar-improving breeding. A new hybrid red currant material was obtained and studied by the method of interspecific hybridization. Correlation analysis was used to assess the relationship between adaptively significant and economical and biological traits. To assess intergenotypic variability, hierarchical clustering was used according to the studied features, which allowed combining three standard methods of multidimensional data analysis. Genotypes adapted to different stressors were identified. The genotypes 271-58-24, 44-5-2, 261-65-19, and ‘Jonkheer van Tets’ were found to have a higher ratio of bound water to free water as compared with the others. Moreover, the genotypes of 271-58-24, 261-65-19, 77-1-47, and ‘Jonkheer van Tets’ were found to have less cold damage during the cold periods. The two most productive genotypes were found to be the genotypes 44-5-2, 143-23-35, and 1426-21-80. A dependence of yield on the beginning of differentiation of flower buds, which led to the abundance of flower inflorescences, was revealed. Rapid restoration of leaf hydration ensured successful adaptation of genotypes to the “temperature shock” of the growing season. The genotypes 271-58-24 and ‘Jonkheer van Tets’ were then observed to be far from the test traits and none of these traits were observed to characterize these two genotypes. The genotypes of 261-65-19 and 77-1-47 were then observed to be characterized by their high stability to *Cecidophyopsis ribis* scores. Genotypes 261-65-19 and 271-58-24, obtained with the participation of ‘Jonkheer van Tets’ as the maternal form, showed sufficient resistance to *Pseudopeziza ribis* and *Cecidophyopsis ribis*. Overall results suggested that the hydration recovery of red currant plants is significantly important for a yield improvement. A new cultivar ’Podarok Pobediteliam (genotype 44-5-2) was obtained that meets the requirements of intensive gardening and is characterized by high adaptability, productivity, and technological effectiveness.

## 1. Introduction

In conditions of climate instability and modern economic conditions, it is important to increase the reliability of agrophytocenoses of berry crops. In order to increase the tolerance of perennial crops to various kinds of stress factors and reduce the level of chemical and technogenic effects on agrocenoses, the priority direction is the accelerated creation of cultivars and hybrids of a new generation with specific resistance, carrying simultaneously several genes of resistance to abiotic and biotic factors, ensuring the technological effectiveness of crop cultivation and high commodity quality of products based on modern methods and improvement of traditional methods (including interspecific and intercultivar hybridization). The demand for most berry crops is currently increasing in world markets. Among berry crops, red currant is very popular, which has become widespread due to the significant demand for berries by virtue of their high therapeutic effect, technological efficiency, and other economically valuable characteristics [1,2]. Most of the known red currant cultivars (‘White Crape’, ‘Versailles Blanche’, ‘Blanka’, ‘Viksne’ and others) show low tolerance to various kinds of stressors when they are introduced into new environments and may not meet the requirements of the modern market. Many researchers emphasize a decrease in the resistance of red currant genotypes to low temperatures; this applies primarily to generative (flower) buds, during the period of sharp changes in daytime and nighttime temperatures at the time of the dormant period [3,4,5,6,7,8,9], as well as reduction of resistance of red currants to higher temperatures of the growing season, soil, and air droughts. A fairly frequent phenomenon is the shedding of the ovary [10,11,12,13,14,15]. Many well-known red currant cultivars have reduced resistance to diseases (*Septoria ribis, Sphaerotheca mors-uvae*) and pests (*Cecidophyopsis ribis*); the absence of resistant genotypes to leaf spots has been reported. This leads to significant yield losses and a reduction in the production area of currants [16,17,18]. Currently, the main industrial assortment of red currants in the USA, Europe, and Asia is represented by the cultivars of European breeding and a small number of old cultivars of Russian breeding: ‘Hollandische Rote’ (‘Prins Albert’), ‘Jonkheer van Tets’, ‘Natal’ ‘Shchedraya’, ‘Rubin’, ‘Rozetta’, ‘Rondom’, ‘Rolan’, ‘Red Pool’, ‘Red Lake’, ‘Rovada’, ‘London market’, ‘Detvan’, and ‘Red Cross’. The use of the interspecific hybridization of red currants for adaptability gives the possibility of creating ecologically balanced agroecosystems with high and stable yields.

In Russia, the red currant cultivar ‘Chulkovskaya’ is used as a source of early ripening, winter hardiness, productivity, and resistance to American powdery mildew. On its basis, early-ripening cultivars ‘Niva’ (Minnesota × Chulkovskaya), ‘Asya’ (Chulkovskaya × Maarses Prominent), and selected seedlings 143-23-25 (Chulkovskaya × Jonkheer van Tets) and 44-5-30 (Chulkovskaya × Minnesota) were developed, which mature simultaneously with ‘Jonkheer van Tets’ and have the same complex of adaptively significant traits. To create super-early and productive cultivars, ultra-early ‘Skorospelaya’ (‘Rannyaya Favorskoy’), a descendant of *Ribes palczewskii* (Jancz.) Pojark, is involved in breeding. The selected seedling obtained on its basis (1097-25-118) ripens 1–1.5 weeks earlier than ‘Jonkheer van Tets’. In the creation of adaptive, long-leaved cultivars, ‘Rote Spatlese’, a descendant of *Ríbes multiflórum*, is used. European cultivars such as ‘Primus’, ‘Rozetta’, ‘Rovada’ and ‘Detvan’ were created on the basis of this cultivar [1,19,20,21].

The effectiveness of breeding work is largely determined by the presence, correct assessment, and selection of initial forms that have a high level of valuable traits and transmit these traits to their offspring. The genetic resources of currants and their wild relatives is one of the important components of biodiversity and serves as an initial material for identifying sources and donors, on the basis of which highly productive, adapted cultivars are created [22,23]. *Ribes* breeding programs are based on interspecific crosses using cultivars and selected forms of different genetic origin, such as poly- and oligogenic sources, as well as donors to *Sphaerotheca mors-uvae* (genes *M1-3, R, Sph2-3*), leaf spot (genes *Pr1* and *Pr2*), and gall mite (genes *Ce* and *P*), as well as other important economically valuable traits, such as bush straightness (*Co*), internode length (*In*), self-fertility, drought resistance, winter hardiness, stable yield, etc. [24,25,26]. The purpose of this study was to obtain new genetic material of red currants on the basis of intercultivar and interspecific crosses (combinative breeding) of different genetic origin, to assess and isolate genotypes adapted to destructive environmental factors in laboratory and field conditions (heat resistance and winter hardiness), as well as to diseases and pests for use in berry breeding programs and creation of highly technological cultivars for farms.

## 2. Results

### 2.1. Heat Resistance of Genotypes 

**Figure 1:**
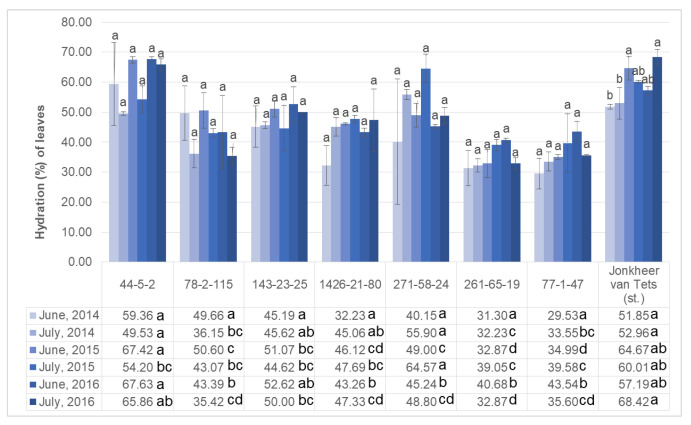
Hydration ability of leaves of selected red currant genotypes during growing period. (Letters above the columns are used to compare growing periods separately for each genotype; and letters next to the values in figure table are used to compare genotypes separately for each growing period. Different letters shown represent significant difference among the values according to Tukey’s HSD test).

High air temperature is an important component of drought [27,28,29]. The water regime is one of the components of plant adaptation to growing conditions. Violations of the water balance leads to overheating of plants to lethal temperatures, which must be taken into account when developing agrotechnical measures for growing plants [30,31]. In this experiment, red currant plants were artificially (under laboratory conditions) subjected to extreme temperatures during important periods of development: growth and ripening of berries. The hydration potential of the selected red currant genotypes was determined in the current study. The results are presented in Figure 1. Although the hydration potential of ‘Jonkheer van Tets’ and genotype 44-5-2 are higher than the other genotypes, no significant difference was noted in June 2014. This difference was then noted as statistically significant in July 2014. The overall hydration of the red currants increased in time, when comparing 2014, 2015, and 2016, but this increase was also noted to be non-significant. The overall results suggested that the genotype 44-5-2 has similar hydration potential with ‘Jonkheer van Tets’. These two genotypes have higher than 50% hydration potential and can be accepted as more resistant to heat stress. 

**Figure 2:**
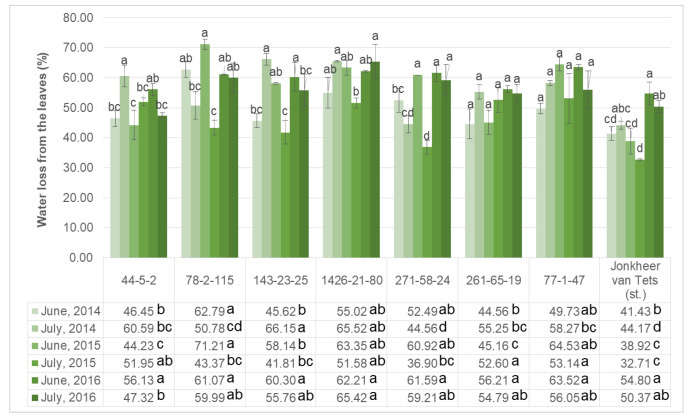
Water loss from the leaves of selected red currant genotypes during growing period. (Letters above the columns are used to compare growing periods separately for each genotype; and letters next to the values in figure table are used to compare genotypes separately for each growing period. Different letters shown represent significant difference among the values according to Tukey’s HSD test).

Heat resistance of the crops is also strongly related with the water loss from the leaves and shoots too. Results suggested that the water loss of the leaves change according to both growing period and genotypes (Figure 2). Three-year studies have shown that throughout the growing season, the genotypes obtained on the basis of ‘Chulkovskaya’—44-5-2 and 143-23-25, as well as the control cultivar ‘Jonkheer van Tets’, have increased heat resistance. Genotypes obtained with the participation of ‘Rote Spatlese’ have a low heat resistance potential, since they lose a lot and weakly restore hydration. Two of the genotypes (78-2-115 and 271-58-24) showed higher water loss in June, while the other showed higher water loss in July in 2014. In June (2014, 2015 and 2016) and July (2014 and 2015) the lowest water loss from the leaves was observed from ‘Jonkheer van Tets’. The only difference was observed in the final measurement point in July 2016 and the lowest water loss was noted from genotype 44-5-2. However, no significant difference was observed between ‘Jonkheer van Tets’ and genotype 44-5-2. On the other hand, the genotypes 78-2-115, 1426-21-80, and 77-1-47 were found to have the highest water loss from the leaves, which is the sign of less resistance against heat stress conditions. 

### 2.2. Flowering and Fruiting of the Genotypes 

The relationship of the data obtained in the laboratory was compared with the field assessment of genotypes and was considered as a variant of drought resistance in natural conditions. The studied genotypes during many years of research have shown optimal resistance to short-term droughts of the growing area. Flowering scores of the genotypes show a slight change during the growing years. The responses of genotypes to different growing seasons were also found to be different (Figure S1). The genotype 271-58-24, 261-65-19, and ‘Jonkheer van Tets’ showed the most stable flowering in different growing years. However, among the test genotypes, the best performance in terms of flowering was observed from genotype 1426-21-80. 

Fruiting scores of the genotypes were also found to be similar with the flowering scores (Figure S2). Ovary shedding in some genotypes during the hot periods of the spring-summer period took place and reached 20% per bush in individual genotypes (2014), 20–30% (2015), and 10–15% (2016), however, there was no severe damage to yield (Supplement 2). Likewise, the flowering, same genotypes (271-58-24, 261-65-19, and ‘Jonkheer van Tets’) showed no significant difference among the years in terms of fruiting. The highest fruiting scores were also obtained from the same genotypes of flowering, the genotype 1426-21-80. 

### 2.3. Yield of the Selected Genotypes

Yield is the most important outcome of all parameters, where all combine and impact the fruit yield of the plants. The yield of the red currant genotypes in the present study was found to have a significant change during the growing seasons. The most productive years were recorded as the year 2016 and year 2018 (Figure S3). In year 2016, the highest yield was noted from the genotype 1426-21-8 with 22.38 t/ha and followed by genotype 44-5-2 with 18.90 t/ha and genotype 143-23-25 with 14.70 t/ha. In the other productive year (2018), the top genotype of year 2016 (1426-21-80) was found to give the lowest yield with 7.00 t/ha. In this year, the highest yield was obtained from the genotype 44-5-2 with 19.60 t/ha and followed by genotype 143-23-35 with 16.80 t/ha. These two genotypes were noted to be the most stable genotypes and growing year had less impact on the yield of these two genotypes. 

### 2.4. Disease and Pest Tolerance of the Genotypes 

The disease infections scores of *Sphaerotheca mors-uvae* ((Schw.) Berk et Curt.) were presented in Figure S4. Among the genotypes, the 44-5-2 and 261-65-19 were observed to be the most tolerant genotypes against *S. mors-uvae* while they got the lowest disease infection scores. During 2016–2018, there was a tendency to increase the infectious load at the site of the primary variety study of currants and, accordingly, the indicators of infection with this disease increased and depended on the growing conditions. Optimal temperature and heavy precipitation led to an outbreak of the mycelium of the fungus *Sphaerotheca mors-uvae*, at the same time, drought against the background of unstable, short-term precipitation provoked severe damage to the leaves of red currant *Pseudopeziza ribis* Kleb (Figures S4 and S5). Therefore, we focused on the search for genotypes with complex resistance to these diseases.

The genotypes of 143-23-25, 1426-21-80, and 77-1-47 were found to be very sensitive and mostly affected by both *S. mors-uvae* and *Pseudopeziza ribis* Kleb. The most resistant genotype against *P. ribis* was observed as the ‘Jonkheer van Tests’. Moreover, the disease infection of *P. ribis* at the genotypes 44-5-2-, 78-2-115, 271-58-24, and 261-65-19 was found to be lower than the score 1.5 during the growing years. 

Besides the above-discussed diseases, the pest of *Cecidophyopsis ribis* West. is also significantly impacting the red currant crops. At the same time, a high percentage of infection in 2016–2018 was associated with the use of a combine harvester during harvesting, which led to the maximum spread of the pest on the site, and here such a fact as genotypic resistance was important, as well as low temperatures of the dormant period, which led to a certain percentage of mite death. The genotypes of 143-23-25 and 77-1-47 were observed to be the most sensitive genotypes against this pest (Figure S6). The other genotypes were found to be tolerant against the *C. ribis* damages, where less damage was observed during the growing periods. 

### 2.5. Cold Resistance of Genotypes 

The hardiness of berry crops is determined by the ability not only to withstand low temperatures, but also to maintain resistance to sudden temperature fluctuations during dormancy (thaw and a sharp drop in temperature during the day).

**Figure 3:**
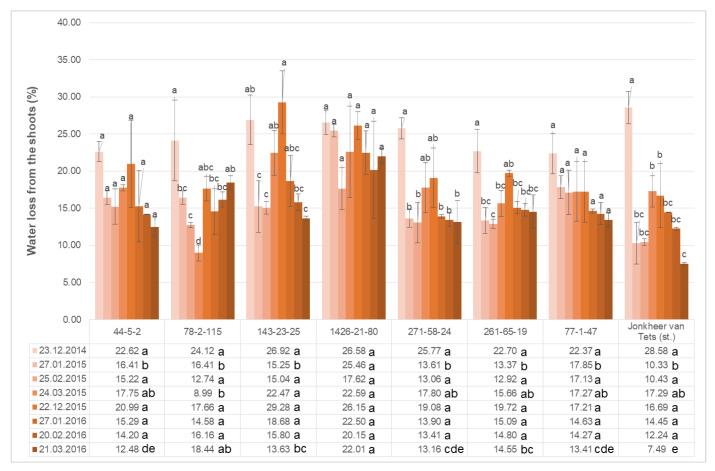
Water loss from the shoots of selected red currant genotypes during dormant period. (Letters above the columns are used to compare growing periods separately for each genotype; and letters next to the values in figure table are used to compare genotypes separately for each growing period. Different letters shown represent significant difference among the values according to Tukey’s HSD test).

Water loss from the shoots during the dormant period is also very important in terms for estimates of the cold tolerance. Results suggested that the water loss from the shoots is significantly varying among the genotypes and also among the periods (Figure 3). The water loss from the shoots is significantly higher at December in each year and shows a decline in other months (January, February, and March). However, this variation was found to be non-significant for some genotypes (44-5-2, 1426-21-80 and 77-1-47). From December to February, the differences between the studied genotypes were not significant, which could be explained by the genetic origin and growing conditions of the species forms and cultivars on the basis of which they were obtained. These months (January and February) are cooler than March. Therefore, it can be suggested that the water loss from the shoots of the genotypes is the same during the cold periods and significantly different during warmer months. It is in March that frequent thaws against the background of sharp low night temperatures are one of the causes of damage to flower buds, annual shoots, and ultimately lead to a decrease in resistance to a complex of factors and crop loss. Minimal water losses in March provide a certain resistance to abiotic stressors of the winter period. In the month of March, the genotype 1426-21-80 was noted to have the highest water loss, while the ‘Jonkheer van Tets’, 78-2-115 had the lowest water loss from the shoots. 

### 2.6. Fractional Water Composition of the Genotypes 

**Figure 4:**
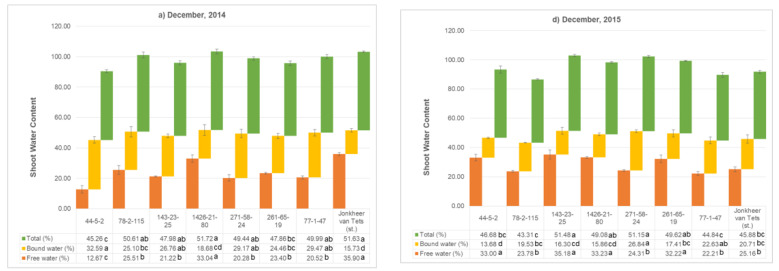

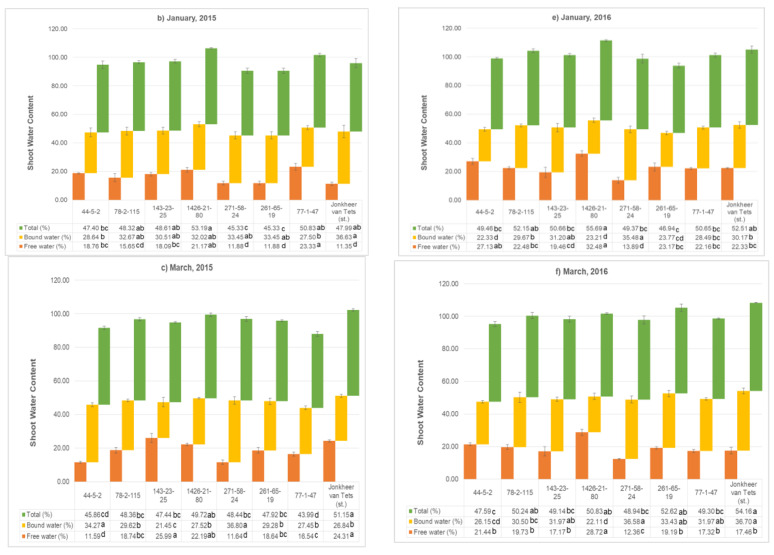
Shoot water content (free water, bound water, and total; %) of the shoots of selected genotypes during dormant period. (Letters next to the values in figure table are used to compare genotypes separately for measured parameters. Different letters shown represent significant difference among the values according to Tukey’s HSD test).

Fractional water composition, both free and bound water, are very crucial for understanding the cold hardiness of the plants during the dormant period. The amount of free water in plant tissues is crucial for regular physiological processes and the bound water is so significant and has positive correlation with the plant’s resistance to unfavorable environmental conditions, including cold [29]. For this reason, the free water and bound water of the red currant genotypes were measured in December, January, and March during 2014–2016 (Figure 4). In December 2014, the ‘Jonkheer van Tets’ and genotype 1426-21-80 were found to have highest free water content and lowest bound water content. Moreover, genotype 44-5-2 was noted to have the highest bound water content and followed by the genotype 77-1-47 and genotype 271-58-24. On the other hand, the content of bound water decreased in December 2015 at the same genotypes. Among all, genotype 271-58-24 was the only one having almost equal content of bound water in both years. The bound water content of the genotype increased in January and then showed a slight decrease in March. Among these months, January is the coldest month, followed by December and March. It can be concluded from the results that the decrease in temperatures results with an increase in the bound water. In other words, the increase in the bound water content of the genotypes helps the plants to resist cold temperatures. 

The ratio of bound water to free water is also an important indicator to understand the cold hardiness characteristic of the genotypes. This ratio was found to have a significant difference both among the genotypes and dormant periods. As mentioned above, the coldest month was January. The evaluation of the results of both 2015 and 2016 showed that the genotype 271-58-24 is the best in terms of the highest ratio of bound water to free water (Figure S7). This genotype was followed by genotype 44-5-2, genotype 261-65-19, and ‘Jonkheer van Tests’. 

### 2.7. Cold Hardiness of the Genotypes 

**Figure 5:**
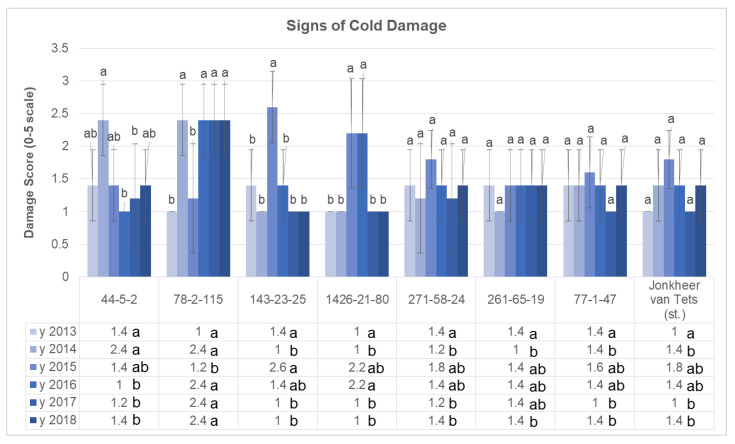
Cold resistance scores of the selected genotypes. (Letters above the columns are used to compare years separately for each genotype; and letters next to the values in figure table are used to compare genotypes separately for each year. Different letters shown represent significant difference among the values according to Tukey’s HSD test).

The data of laboratory assessment of genotypes to low temperatures were compared with field observations. Cold damage scores of the genotypes were all determined by using the 0–5 scale (Table S1). Results suggested that the cold damage of the genotypes vary from 1.0 to 2.6 (Figure 5). According to the results obtained, the cold damages of the genotypes of 271-58-24, 261-65-19, 77-1-47, and ‘Jonkheer van Tests’ had no significant difference in different years. On the other hand, the cold damage of the other four genotypes (44-5-2, 78-2-115, 143-23-25, and 1426-21-80) showed significant increase in some years, especially in 2014 and 2016. The climatic data of the experimental site shows that these 2 years were the coolest years of the study period. Therefore, results of current study are logical, while the decrease in temperature caused an increase in the cold damage of some genotypes. 

### 2.8. Clustering and Genotype Similarity 

**Figure 6:**
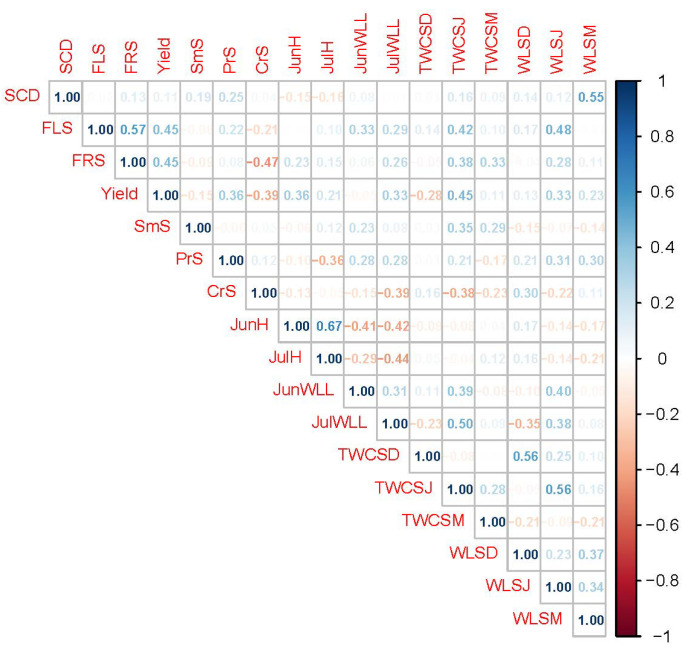
Correlation among biometric parameters, heat-resistant characteristics, and water regime traits of plant leaves and shoots of red currant plants. Abbreviations: SCD: Signs of Cold Damage; FLS: Flowering Scores; FRS: Fruiting Scores; Yield: Yearly Yield; SmS: *Sphaerotheca mors-uvae* Score; PrS: *Pseudopeziza ribis* Score; CrS: *Cecidophyopsis ribis* Score; JunH: Hydration of leaves in June; JulH: Hydration of leaves in July; JunWLL: Water loss of leaves in June; JulWLL: Water loss of leaves in July. TWCSD: Total water content of shoots in December; TWCSJ: Total water content of the shoots in January; TWCSM: Total water content of the shoots in March; WLSD: Water loss from shoots in December; WLSJ: Water loss from shoots in January and WLSM: Water loss from shoots in March.

Most of the study parameters of red currant genotypes were found to have a negligible amount of positive or negative correlations (Figure 6). The highest positive was observed between the hydration of leaves in June and hydration of leaves in July. The correlation score between these parameters was 0.67. On the other hand, a moderate correlation (0.57) was also noted between the flowering scores and fruiting scores. Besides that, water loss from shoots in January was found to have a moderate positive correlation (0.56) with total water content of shoots in December. Same correlation (0.56) was recorded between the water loss from shoots in January and total water content of the shoots in January. One of the important correlations was observed between the water loss from shoots in March and signs of cold damage (0.55). The highest negative correlation was recorded as −0.47 between the fruiting score and C. ribis scor, which clearly describes that the pest damage reduces the fruiting of the red currant genotypes. Similarly, this pest was noted to have a −0.39 correlation with the yield. 

**Figure 7:**
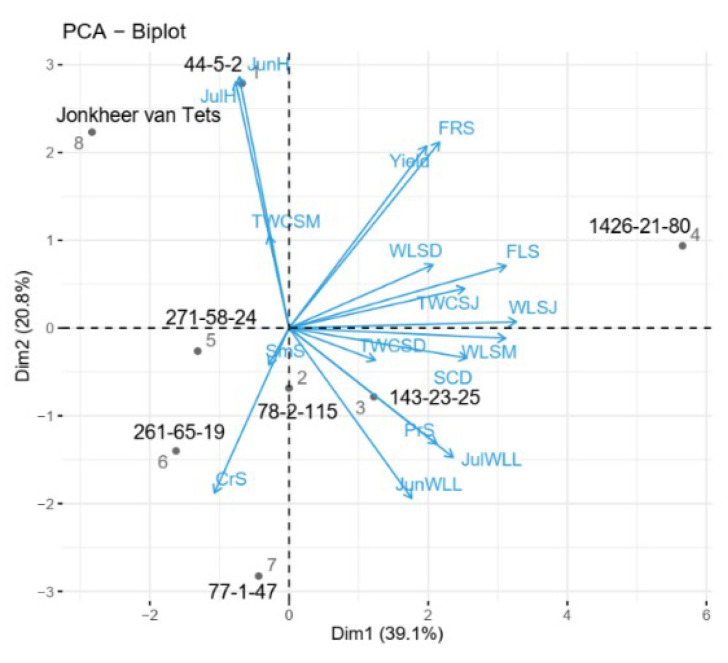
PCA—biplot with loadings analysis of red currant genotypes. Abbreviations: SCD: Signs of Cold Damage; FLS: Flowering Scores; FRS: Fruiting Scores; Yield: Yearly Yield; SmS: *Sphaerotheca mors-uvae* Score; PrS: *Pseudopeziza ribis* Score; CrS: *Cecidophyopsis ribis* Score; JunH: Hydration of leaves in June; JulH: Hydration of leaves in July; JunWLL: Water loss of leaves in June; JulWLL: Water loss of leaves in July. TWCSD: Total water content of shoots in December; TWCSJ: Total water content of the shoots in January; TWCSM: Total water content of the shoots in March; WLSD: Water loss from shoots in December; WLSJ: Water loss from shoots in January and WLSM: Water loss from shoots in March.

The impacts of the test traits on the genotypes was shown in Figure 7. Results demonstrated that the genotype 44-5-2 was mostly characterized by hydration of leaves in June and July. The genotypes 271-58-24 and ‘Jonkheer van Tets’ were then observed to be far from the test traits and none of these traits was observed to characterize these two genotypes. The genotypes of 261-65-19 and 77-1-47 were then observed to be characterized by their high *C. ribis* scores. The genotype 78-2-115 was also found to have high *C. ribis* scores and highly characterized by its high water loss from leaves in July. The genotypes 1426-21-80 and 143-23-25 were finally observed to be affected from several traits at the same time. However, the most important characteristic of genotype 1426-21-80 was its flowering score and the genotype 143-23-25 was mostly characterized by its high water loss from leaves in July. 

**Figure 8:**
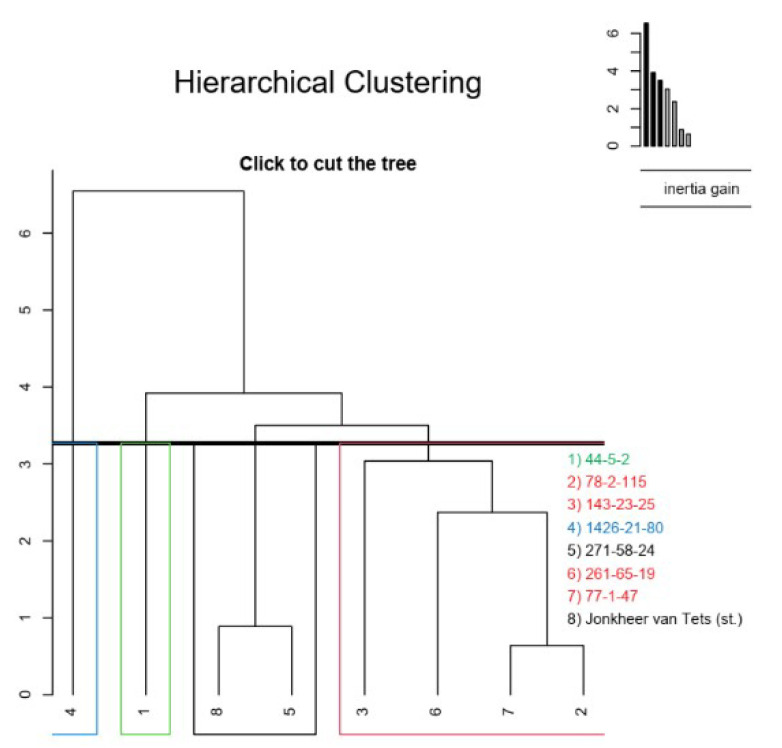
Hierarchical cluster analysis of red currant genotypes.

Hierarchical clustering (Figure 8) suggested four meaningful clusters, where the genotype 1426-21-80 was found alone in a cluster, genotype 44-5-2 in another cluster alone, and genotypes 271-58-24 and ‘Jonkheer van Tets’ in another cluster. The other four genotypes were then grouped in another cluster. 

**Figure 9:**
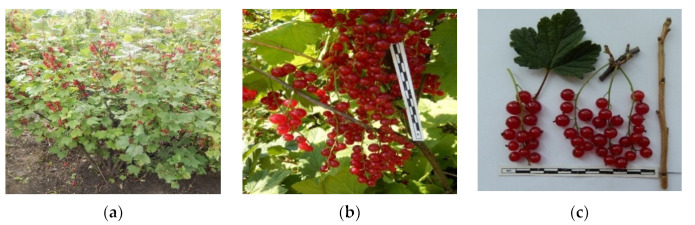
(**a**) Bush habitus; (**b**) fruiting; and (**c**) pomological traits of ‘Podarok Pobediteliam’ (genotype 44-5-2). Selection plot at the Russian Research Institute of Fruit Crop Breeding (VNIISPK).

Based on the conducted studies, 44-5-2 (Figure 9) was identified as the most drought-resistant, heat-resistant, and winter-hardy selection form, having a wide range of morphological and physiological adaptive features (regulation of water forms during the growing season and dormancy, high water-retaining ability of shoots, optimal resistance to diseases and pests, high yield, bush straightness, and the possibility of using combine harvesting) and it was transferred as a ‘Podarok Pobediteliam’ cultivar to the State Commission of the Russian Federation for Testing and Protection of Selection Achievements in the State Register of Selection Achievements for industrial cultivation in Central Chernozem regions of Russia. This cultivar can be used for introduction to the countries of Central and Western Europe, Turkey, USA.

## 3. Discussion

Heat, drought, and low temperatures are among the main abiotic stressors to which agricultural crops are exposed and which lead to large crop losses [27,31,32]. The critical, lethal temperature for *Ribes* is considered to be +50 °C [33], therefore, this temperature was chosen in this experiment to assess the heat resistance of red currant genotypes and should be taken into account when introducing them to other regions. In the period of extreme drought, the key role belongs to the ability to restore a certain percentage of lost water after a temperature shock [34]. Rapid recovery after rehydration indicates the ability of the species, genotypes to cope with destructive factors of the growing season, and successfully adapt to drought conditions. However, the prolonged water stress in garden crops can lead to the disruption of the flow of water to the leaf tissues, and the restoration of water flow to the leaf tissues will not be complete (100%) [35,36,37]. When exposed to short-term heat shock, the studied genotypes showed cultivar specificity. The genotypes did not restore the initial level of hydration. The maximum percentage of damage was in crossing combinations where ‘Rote Spatlese’ was used in parent pairs. A high level of resistance to hyperthermia was shown by genotypes developed with the participation of ‘Chulkovskaya’. In the studies conducted on currant cultivars in different ecological and geographical zones, a different percentage of water recovery was also noted, depending on the duration of exposure to high temperatures, genetic origin, as well as prolonged exposure to “heat shock” led to necrosis of leaf tissues [38,39]. Information about the water status of fruit crops is necessary when planning an irrigation program that will reliably ensure the optimal functioning of physiological indicators. However, at the same time, there is no consensus on a suitable indicator on this issue and the topic remains debatable [40,41,42,43].

In the experiment, the field drought had a different effect on the degree of flowering and variability of the berries of the studied red currant genotypes; the influence of genetic and geographical origin was clearly traced. It is noted that the shedding of the ovary in red currant can reach up to 30–40% of the potential yield. At the same time, even in years with optimal weather conditions, the shedding can be up to 10%. The reasons for the interannual variability of ovary shedding may be weather conditions, physiological and hormonal disorders in plants, incompatible genotypes, biotic factors, and limited resources due to too many fruits. The maximum fall of berries in this experiment was observed at 1–1.5 weeks before biological maturation. The nature of the fall of berries seemed to be quite dynamic and could continue both during the entire growing season (2014) and a short period (2013, 2015). A similar pattern was observed in blueberry species [44]. The search for reports on the patterns and causes of ovary fall in other berry crops turned out to be futile.

The influence of the currant genotype on the shedding of berries during soil and air drought during the flowering and fruiting periods has already been noted in the reports of a number of authors [45,46,47,48,49]. In this experiment, the genotypes developed on the basis of ‘Jonkheer van Tets’ and species growing in mountainous areas (*Ribes meyeri*, *Ribes atropurpureum* C.A. Mey) showed their resistance to drought; shedding of their flowers and berries was minimal. At the same time, a strong relationship between the number of flowers in the inflorescence of red currants and the degree of ovary fall was not revealed in this study. However, a certain factor affecting the decrease in the yield of the studied genotypes of red currants is the age of the bush, the maturation period, and soil moisture. These observations are consistent with the results obtained on black currant and grapes [45,50,51,52,53,54]. In this study, the regularity of the dependence of yield on the successful initiation of differentiation of generative buds (flower buds) is clearly traced, which leads to an abundance of flower inflorescences. With abundant flowering, the fall of flowers and berries does not significantly affect the degree of yield, which explains the results obtained in genotypes 1426-21-80 and 143-23-25. At the same time, an important factor reducing the degree of flowering and, as a consequence, yield, is the effect of abnormal positive temperatures during the dormant period. In this experiment, sharp temperature changes in March 2015–2016 caused a delay or asynchrony of flowering, a low percentage of flowering and pollination, a delay in vegetative development, and a violation of fruit setting in the red currant genotypes 143-23-25 and 77-1-47 in comparison with optimal conditions. At the same time, low negative temperatures during flowering (spring frosts) in 2017 and 2015 led to a certain percentage of flower death in most of the studied red currant genotypes. Spring frosts are quite common in central Europe and are the reason for the decline in the berry yield. The attempts were made to reduce the fall of black currant flowers by spraying with chemicals (Teric 12A23B and DEPEG), or plant growth and development hormones (auxin, gibberellins) were used to restrain the flowering process of garden crops [55]. There are conflicting opinions on the issue of the difference in spring frost resistance of different genotypes. The effect of spring frosts may vary depending on the duration of the frost period, the degree of differentiation of flowers, and the nature of the influence of cold air [45,56,57]. In this regard, one of the important goals of breeding programs for both black and red currants is the breeding of cultivars with later flowering [58,59,60]. In this experiment, genotypes 1426-21-80, 78-2-115, and 44-5-2 maintained high yields in 2015 and 2017 due to the shift of flowering dates to later dates. Changes in the cyclicity of natural and climatic processes, the growing season of berry crops, as well as weakening of the immune system actualize the program of cultivar-improving breeding using genetic resources and modern diagnostic methods. 

The physiological processes underlying acclimatization to abiotic stressors of fruit and berry crops still remain relevant and poorly understood issues [61,62,63]. The water content in the vegetative parts of the shoot and generative buds plays an important role in adapting to a low-temperature stress. A high correlation between the hydration of buds and shoots, the stage of development, genotype, and frost resistance was revealed in *R. nigrum*, but at the same time the opposite role of the presence of water was indicated in ontogenetic development and deacclimation. In this experiment, the influence of the genetic origin of red currant samples on resistance to low temperatures was traced. Based on the indicators of the water regime of the red currant genotypes, the variability of this trait was confirmed during several winter months. Genotypic differences of black currant cultivars in the kinetics (i.e., nature) of acclimatization, and the development of buds and shoots are determined by different rates of deacclimation [50]. The high water content in shoots and vegetative buds is a prerequisite for an early exit from dormancy and the beginning of growth processes in buds (the deployment of leaf plastics, since in this case a high turgor is necessary) [64]. Winter hardiness and water content are determined by the proportion of bound and free water. A high proportion of bound water ensures adaptation to the lowest possible temperatures [62,63,65]. In this experiment, a relationship is traced between the maximum content of bound water in shoots; high water retention capacity; and a small percentage of damage to shoots and generative and vegetative buds of red currants by negative temperatures in the field in the genotypes, obtained on the basis of ‘Jonkheer van Tets’ and ‘Chulkovskaya’. The antagonistic role of water in the resistance of fruit crops to low-temperature shock and plant development (ontogenesis) may not be accurate enough, because the increased water content in cells is accompanied by cell growth and is one of the causes of mechanical intercellular damage [66,67,68,69]. 

Interspecific hybridization of *Ribes* is widely used in the introduction of resistance genes to biotic stressors. Many scientists have shown the prospects of involving the red currant cultivar ‘Jonkheer van Tets’ for breeding for resistance to *Sphaerotheca mors-uvae, Pseudopeziza ribis,* and *Cecidophyopsis ribis* [70,71,72,73]. In this study, the genotypes 261-65-19 and 271-58-24 obtained with the participation of ‘Jonkheer van Tets’ as the maternal form showed sufficient resistance to *Pseudopeziza ribis* and *Cecidophyopsis ribis.*

## 4. Materials and Methods

The studies were carried out during the growing season and dormancy periods of plants during 2013–2018 at the Russian Research Institute of Fruit Crop Breeding (VNIISPK) at the site of the primary variety study of red currants.

### 4.1. Experimental Site and Setting Up the Experiment

**Figure 10:**
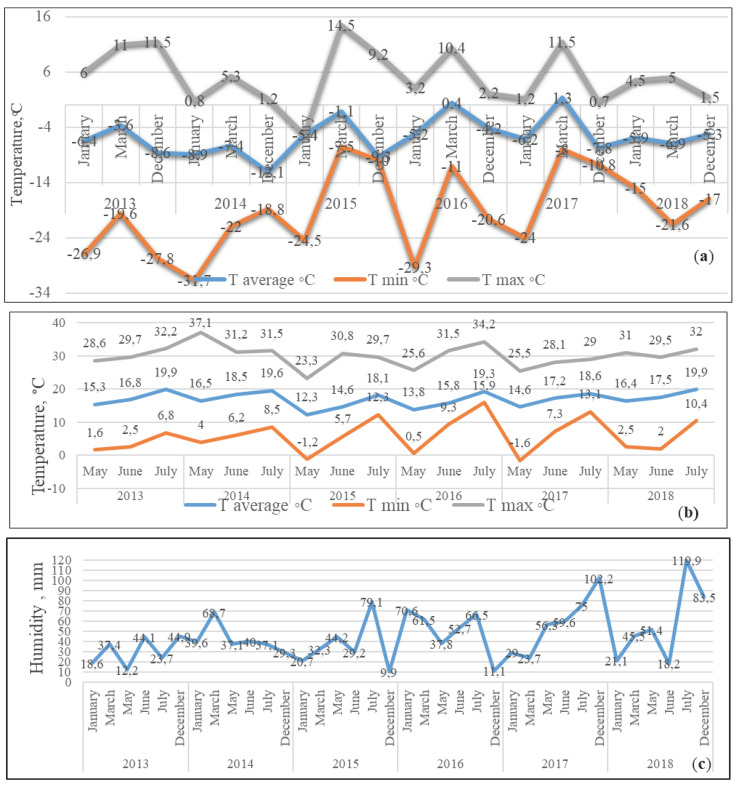
(**a**) Monthly average, minimum, and maximum air temperatures during: (**b**) dormancy and (**c**) growing period, and the amount of precipitation during dormant and growing seasons at the Zhilina, Orel District, Orel Region, and Russian Federation.

The site is located in a zone with a temperate continental climate (52°96’ north latitude; 36°07’ east longitude). Winter is moderately cool. Periodic cold spells are replaced by thaws. Summer is unstable, with alternating periods of heat and coolness. Atmospheric precipitation falls in moderate amounts and is distributed extremely unevenly over the months. The climatic data about temperatures and humidity of the experimental site was shown in Figure 10. The air temperature during the dormancy period was characterized by sharp changes in daytime and nighttime temperatures; short-term thaws were frequent. For the central region of Russia, the optimal snow cover was established only by the 3rd day of December, while at the beginning and middle of this month, very low temperatures (2013, 2014, and 2016) and short-term thaws were frequent, which led to an imbalance of the protective mechanisms of plants. Winters in 2014, 2015, and 2016 were the coldest ones. In March, short-term changes in night and daytime temperatures led to damage to generative buds and annual shoots. During the growing season, there were separate periods with optimal temperature and humidity, as well as dry ones. The most unfavorable conditions for plant growth and development were in May–July 2013, 2014, and 2015, and May 2016. In May 2015 and 2017, negative night temperatures were observed, which led to a certain percentage of generative bud damage in individual genotypes. 

The soil of the site was loamy haplic luvisol. The thickness of the humus horizon is 30–55 cm. Some important agrochemical indicators of experimental soil, determined by standard methods [74], are presented in Table 1. 

Scheme of planting experimental plants was 2.8 × 0.5 m. The soil in the aisles was kept under black steam and was not irrigated. The soil in the rows of shrubs during the entire research period was kept under the Roundup herbicide (active ingredient C_3_H_8_NO_5_, 360 g × L^−1^). Mineral fertilizers (ammonium nitrate (NH_4_NO_3_) were applied once a year in early April, in an amount of 50–60 g for each plant throughout the experiment. Treatment of all plants from diseases and pests was carried out after their accounting, with infection of more than 30% of the experimental site. Penconazole (C_13_H_15_C_l2_N_3_) was used against *Sphaerotheca mors-uvae*; Malathion (C_10_H_19_O_6_PS_2_) was used against pests (*Criptomyzus ribis* L., *Geometridae*, *Cecidophyopsis ribis*).

### 4.2. Red Currant Breeding, Selection of Genotypes

Intercultivar and interspecific crosses were carried out in early May 2007. According to phenotypic characteristics, the following cultivars were selected from the genetic collection of red currants as the initial parent pairs:

- ‘Rote Spatlese’—a winter hardy cultivar resistant to leaf spots and medium stable to *Sphaerotheca mors-uvae* and *Cecidophyopsis ribis*; ‘Chulkovskaya’—a drought stable cultivar with high self-fertility, medium winter hardiness, and medium resistance to *Sphaerotheca mors-uvae* and *Cecidophyopsis ribis*;
- ‘Maarses Prominent’—a winter hardy fruitful cultivar with high self-fertility and resistance to fungal diseases (*Sphaerotheca mors-uvae*) and pests *Cecidophyopsis ribis* and *Septoria ribis*;
- ‘Minnesota’—a drought resistant and productive cultivar having large berries, medium winter hardiness and medium resistance to *Sphaerotheca mors-uvae*, and pests *Cecidophyopsis ribis* and *Septoria ribis*; Ribes meyeri—a winter hardy red currant species with high yields and high resistance to diseases and pests;
- *Ribes atropurpureum* C.A. Mey—a winter hardy red currant species with high yields and high resistance to diseases and pests;
- ‘Jonkheer van Tets’—a winter hardy cultivar having high yields, resistance to the main diseases and pests, and medium stability to drought.
- Red currant cultivars and species used in hybridization were obtained from various Russian Research Institutes (‘I. V. Michurin Federal Scientific Center’, ‘Central Siberian Botanical Garden ‘, ‘Federal Research Center N. I. Vavilov All-Russian Institute of Plant Genetic Resources’ (VIR), ‘Federal Scientific Breeding and Technological Center for Horticulture and Nursery’).

**Figure 11:**
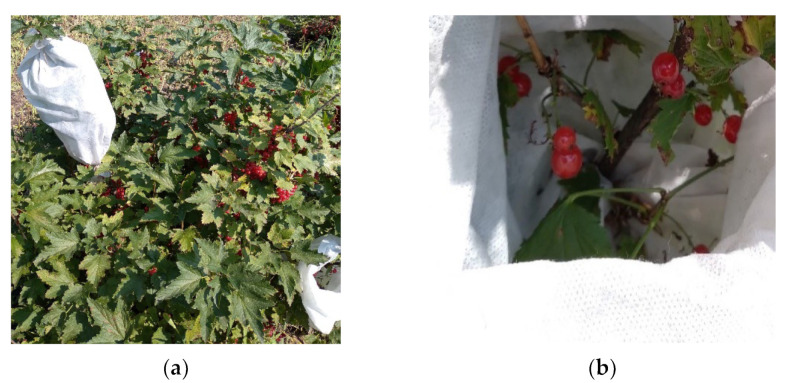
(**a**) Isolation bags on the maternal form of red currants and (**b**) berries obtained as a result of intercultivar hybridization of ‘Jonkheer van Tets’ cultivar. The site of the primary variety study of red currants at the Russian Research Institute of Fruit Crop Breeding.

Hybridization was performed using castration of flowers from the maternal plant of a certain cultivar, no more than 50 flowers in one repetition, and application of pollen of the paternal form, followed by isolation of these shoots. We took into account the fact that disease resistance was better tolerated on the maternal side, which was associated with the cytoplasmic localization of part of the resistance genes. The berries were taken from the bags at the end of July, as a rule, no more than 15 berries were obtained from one bag. Each cultivar had six bags (six repetitions) (Figure 11).

Seeds were extracted from the berries and stratified in *Sphagnum moss* at a temperature of +2 ± 5 °C within 20 days. Sowing of seeds was carried out in early September. During 2008–2009, the genotypes of red currant were studied according to a complex of economically valuable traits: resistance to diseases and pests; field assessment of the condition of plants after winter. In 2010, seven seedlings with varying degrees of resistance to biotic and abiotic factors were selected from that group of plants (Table 2). These seedlings were transferred to the site of the primary variety study of red currant for further study. As a control, ‘Jonkheer van Tets’ was selected, which had been included by the State Commission of the Russian Federation for Testing and Protection of Selection Achievements in the State Register of Selection Achievements for industrial cultivation in the North-Western, Volga-Vyatka, and Central Chernozem regions of Russia.

### 4.3. Determination of Stability Characteristics of Selected Genotypes 

To assess the stability of genotypes during the growing and dormant periods, laboratory experiments were conducted. For each analysis, five annual shoots and/or leaves from one red currant genotype were used in the below described analysis.

#### 4.3.1. Assessment of the Hydration Ability of Genotypes 

The hydration (ability to absorb water) of the leaves was assessed during June and July in 2014, 2015, and 2016. A climate chamber ‘Espec’ PSL—2KRN (ESPEC, Osaka, Japan) was also used to simulate drought conditions (heat resistance). The heat resistance of the leaves was assessed by the degree of restoration of their hydration after a temperature shock of +50 °C for 30 min. The degree of restoration of hydration was determined by weighing samples of leaves after wilting (exposure time 2 h, temperature +22 ± 25 °C) followed by saturation with water for 24 h. Analytical scales Discovery DV 114C (OHAUS, Parsippany, NJ, USA) with an accuracy of 0.01 mg was used. The percentage of hydration recovery was calculated as follows (1):

(1) $$DW=\frac{b2-b1}{b0}\cdot 100\%,$$

where *DW* is water absorption at saturation in percent of lost water [75], *b*_2_—weight of leaves after saturation with water (g); *b*_1_—weight of leaves after wilting (g); *b*_0_—weight of water lost by the leaf (g).

#### 4.3.2. Determination of the Water-Holding Capacity of the Genotypes 

The water-holding capacity of the leaves and shoots was determined by the amount of water lost by the leaves during growing period (June and July) in 2014, 2015, and 2016; and by the shoots during dormant periods (December–March) in 2015 and 2016. The shoots kept at a temperature of +25 °C for 12 h before analysis. The calculation was carried out according to the Formula (2) [76]. The mass of shoots was weighed on laboratory scales Scout Pro SP 202 (OHAUS, Parsippany, NJ, USA).

(2) $$WHS=\frac{m2-m1}{m0}\cdot 100\%,$$

where *WHS* is amount of water lost by the shoots (%); *m*_1_—initial weight of shoots (g); *m*_2_—weight after wilting at a temperature of +25 °C (g); and *m*_0_—absolutely dry weight of the shoot after drying at a temperature of +105 °C (g).

#### 4.3.3. Determination of Fractional Composition of Water of the Genotypes 

The fractional composition (free water and bound water) of water plant shoots was determined by the refractometric method, by following the method of Okuntzova-Marinchik [77]. The method is based on a change in the concentration of a sucrose solution when a shoot sample is immersed in it. Test tubes with stoppers were used. Two milliliters of 30% sucrose solution was used for this purpose. The average sample weight was 400 mg. Using a PAL 1 refractometer (ATAGO, Tokyo, Japan), the final concentration of sucrose solution was determined after immersion of a plant sample in it (exposure time—2 h). At the same time, the total water content was determined by drying the plant sample (for bound water determination) to a constant weight in an artificial climate chamber ‘Espec’ PSL—2KRN (ESPEC, Osaka, Japan) at 105 °C for 3 h. The total water content (%) of the leaves was then calculated by taking the sum of free and bound water percentages. 

#### 4.3.4. Biometric Evaluation of the Genotypes 

The field assessment of winter hardiness was evaluated after the plants came out of deep dormancy (late March–early April) by damage to shoots (annual and perennial), and vegetative and generative buds. The degree of damage was counted in points equivalent to the percentage of damage (Table S1). Five plants of the same genotype were taken into account.

The resistance of red currant genotypes to abiotic stressors during the growing period was assessed in June and July. Five leaves were taken from the middle part of the bush in threefold repetition. Field assessment of resistance to abiotic factors of the growing season was carried out according to the degree of flowering, fruiting, and shedding of berries (Table S2). Five plants of each genotype were taken into account.

The assessment of resistance to diseases (*Sphaerotheca mors-uvae* (Schw.) Berk et Curt.) and leaf spots (*Pseudopeziza ribis* Kleb) and pests (*Cecidophyopsis ribis*) was carried out during the growing season in points equivalent to the percentage of damage (Table S3). Ten plants of each genotype were taken into account for this evaluation. 

#### 4.3.5. Assessment of the Yield of the Genotypes 

Harvest accounting was carried out from 1 July to 10 July by weight, from three accounting plants of each genotype. Initially, the yield from the bush was determined in kilograms, and then recalculated in t ha-1 for each genotype. 

#### 4.3.6. Statistical Data Analysis 

Statistical analysis of the obtained data was carried out by variance analysis (ANOVA) using SPSS, version 22.0, and Microsoft Excel software programs. The relationship of the features was evaluated using correlation analysis. Principal component analysis (PCA) was applied between the studied parameters of genotypes (similarities and differences). To assess intergenotypic variability, hierarchical clustering was used according to the studied features, which allowed combining three standard methods of multidimensional data analysis [78]. The ‘FactoMineR’ and ‘factoextra’ packages in R 4.0.3. (Free Software Foundation’s GNU General Public License) were used for hierarchical clustering on principal components (HCPC), PCA, and correlations analysis.

## 5. Conclusions

The genotypes created on the basis of ‘Jonkheer van Tets’ and ‘Chulkovskaya’ showed high adaptation to abiotic stressors (low temperatures, soil and air drought, maximum high temperatures). The rapid restoration of hydration of red currants during the growing season ensured optimal yield and optimal laying of generative buds for next year’s harvest. The correlation analysis method revealed the dependence of yield on the beginning of differentiation of flower buds. There was no high correlation between the number of flowers in the inflorescence of red currants and the degree of ovary fall. The decrease in yield during the growing season was determined by the age of the bush, the timing of maturation, and soil moisture. Sharp fluctuations in spring temperatures caused asynchronous flowering, a low percentage of flowering and pollination, a delay in vegetative development, and a violation of fruit setting. The late flowering periods of red currants ensured the avoidance of abnormal low temperatures and the preservation of generative buds and yields. The maximum content of bound water in the shoots and high water retention capacity minimized damage to shoots and generative and vegetative buds by negative temperatures and ensured optimal functioning and development of plants during the growing season. Genotypes developed on the basis of ‘Jonkheer van Tets’ as the maternal form showed the resistance to *Sphaerotheca mors-uvae*, *Pseudopeziza ribis,* and *Cecidophyopsis ribis*. By the method of hierarchical clustering, eight red currant genotypes of different genetic origin were grouped into four clusters according to the set of adaptively significant traits. ‘Podarok Pobediteliam’, an adaptive, productive, competitive, technological cultivar, was created for introduction into different ecological and geographical zones.

## Figures and Tables

**Table 1:** Agrochemical indicators of the experimental soil.

Depth (cm)	pH_KCl_	Organic Matter (%)	Content (mg kg^−1^)	
			K_2_O	P_2_O_5_
0–20	5.40 ± 0.15	2.14 ± 0.08	182.50 ± 62.75	208.74 ± 27.60
20–40	5.50 ± 0.20	1.85 ± 0.06	125.42 ± 38.65	149.12 ± 24.34

**Table 2:** Genetic origin of the selected red currant genotypes.

Genotype	Genetic Origin
77-1-47	Rote Spatlese × ‘Jonkheer van Tets’
78-2-115	Rote Spatlese × Maarses Prominent
1426-21-80	(Rote Spatlese × Chulkovskaya) × (Rote Spatlese × Maarses Prominent)
44-5-2	Chulkovskaya × Minnesota
143-23-25	Chulkovskaya × ‘Jonkheer van Tets’
261-65-19	‘Jonkheer van Tets’ × *Ribes atropurpureum* C.A. Mey
271-58-24	‘Jonkheer van Tets’ × *Ribes meyeri*
‘Jonkheer van Tets’ (st.)	Faya Plodorodnaya × London Market

## Data Availability

The data presented in this study are available upon request from the corresponding author.

## Supplementary Materials

The following supporting information can be downloaded at: https://www.mdpi.com/article/10.3390/plants11060802/s1: Figure S1. Flowering scores of the selected genotypes. (Letters above the columns are used to compare years separately for each genotype; and letters next to the values in figure table are used to compare genotypes separately for each year. Different letters shown represent significant difference among the values according to Tukey’s HSD test). Figure S2. Fruiting scores of the selected genotypes. (Letters above the columns are used to compare years separately for each genotype; and letters next to the values in figure table are used to compare genotypes separately for each year. Different letters shown represent significant difference among the values according to Tukey’s HSD test). Figure S3. Yield of the selected genotypes from 2013 to 2018. (Letters above the columns are used to compare years separately for each genotype; and letters next to the values in figure table are used to compare genotypes separately for each year. Different letters shown represent significant difference among the values according to Tukey’s HSD test). Figure S4. Disease infection scores of selected genotypes as affected by *Sphaerotheca mors-uvae* ((Schw.) Berk et Curt.). (Letters above the columns are used to compare years separately for each genotype; and letters next to the values in figure table are used to compare genotypes separately for each year. Different letters shown represent significant difference among the values according to Tukey’s HSD test). Figure S5. Disease infection scores of selected genotypes as affected by *Pseudopeziza ribis* Kleb. (Letters above the columns are used to compare years separately for each genotype; and letters next to the values in figure table are used to compare genotypes separately for each year. Different letters shown represent significant difference among the values according to Tukey’s HSD test). Figure S6. Pest damage scores of selected genotypes as affected by *Cecidophyopsis ribis* West. (Letters above the columns are used to compare years separately for each genotype; and letters next to the values in figure table are used to compare genotypes separately for each year. Different letters shown represent significant difference among the values according to Tukey’s HSD test). Figure S7. Ratio of bound water to free water content of the shoots of selected genotypes during dormant period (December, January, and March). (Letters above the columns are used to compare growing periods separately for each genotype; and letters next to the values in the figure table are used to compare genotypes separately for each growing period. Different letters shown represent significant difference among the values according to Tukey’s HSD test). Table S1. The scale used for the assessment of cold damage on the genotypes. Table S2. The scale used for the assessment of flowering and fruiting of the genotypes during vegetation period. Table S3. Indicators of diseases and pests of red currant genotypes [15].
