# Supplementary material for: Creation and Use of Highly Adaptive Productive and Technological Red Currant Genotypes to Improve the Assortment and Introduction into Different Ecological and Geographical Zones
Source: Plants (Basel). 2022 Mar 17;11(6):802. doi: 10.3390/plants11060802 (PMC8954894; doi:10.3390/plants11060802)
Supplement: Supplementary file 1 [file plants-11-00802-s001.zip › Supplement 10.pdf]

**Table S3.** Indicators of diseases and pests of red currant genotypes

| Score | *Sphaerotheca mors-uvae* | *Pseudopeziza ribis* | *Cecidophyopsis ribis* |
|---|---|---|---|
| 0 | No infection (healthy plant) | No infection (healthy plant) | No damage (healthy plant) |
| 1.0 | Very weak disease damage (up to 10% of leaves and up to 1% of berries are affected) | Very weak damage (up to 5% leaves are affected) | Damage to single buds (до 5%) |
| 2.0 | Weak plant disease (up to ¼ of the shrub shoots, up to 25% of leaves and up to 3% of berries are affected) | Weak plant damage (up to 10% of the leaves are damaged) | Up to 10% of buds on one plant are damaged by mite |
| 3.0 | Average plant disease (up to ⅓ of the shrub shoots, 26-50% of the leaves and up to 10% of the berries are affected) | Average plant damage (up to 30% of leaves are damaged) | Up to 30% of buds on one plant are damaged by mite 30% |
| 4.0 | Severe plant disease (⅓ to ¼ of the shrub shoots, 51-70% of the leaves and up to 20% of the berries are affected) | Severe plant damage (up to 50% of the leaves are damaged) | Severe damage, from 30 to 50% of buds on one plant. |
| 5.0 | Very severe disease (more than ½ of the shrub shoots, more than 70% of the leaves and more than 20% of the berries are affected) | Very severe damage (more than 50% of the leaves are damaged) | More than 50% of buds on one plant are damaged. |
