# Supplementary material for: Creation and Use of Highly Adaptive Productive and Technological Red Currant Genotypes to Improve the Assortment and Introduction into Different Ecological and Geographical Zones
Source: Plants (Basel). 2022 Mar 17;11(6):802. doi: 10.3390/plants11060802 (PMC8954894; doi:10.3390/plants11060802)
Supplement: Supplementary file 1 [file plants-11-00802-s001.zip › Supplement 8.pdf]

Table S1. The scale used for the assessment of cold damage on the genotypes.

Score	Signs of damage
0	No signs of freezing
1.0	Freezing of the upper part of annual shoots (¼ of their length). The number of frozen annual shoots is not more than 10% and up to 10% of generative buds from one bush, perennial shoots and vegetative buds are not damaged.
2.0	Freezing of no more than 20%-25% of annual increments (up to 1/2 of their length), up to 25% of generative buds from one bush, up to 10% freezing of perennial shoots
3.0	Up to 70% of annual shoots are damaged (more than 1/2 of their length), 25-50% of generative buds are frozen, up to 25% of two-year-old and single (up to 10%) perennial shoots from one bush are frozen
4.0	Total (100%) damage to annual shoots; freezing of generative buds up to 75%; two-year and perennial shoots are damaged up to 75%; bush renewal comes from lower (dormant) buds
5.0	Complete (100%) freezing of the aboveground part of the bush, there is no renewal of the bush
