# Supplementary material for: Creation and Use of Highly Adaptive Productive and Technological Red Currant Genotypes to Improve the Assortment and Introduction into Different Ecological and Geographical Zones
Source: Plants (Basel). 2022 Mar 17;11(6):802. doi: 10.3390/plants11060802 (PMC8954894; doi:10.3390/plants11060802)
Supplement: Supplementary file 1 [file plants-11-00802-s001.zip › Supplement 9.pdf]

Table S2. The scale used for the assessment of flowering and fruiting of the genotypes during vegetation period.

Score	Important adaptive, economical and biological traits
1.0	Plants have a single, weak annual growth (less than 5% per bush), the leaves are deformed, not of a typical shape, lag behind in the timing of the passage of phenological phases (flowering and fruiting), single flowers (up to 10% per bush) and the ovary is almost completely crumbling.
2.0	Plants have up to 10% annual growth, flowering and fruiting are weak (up to 20%) on individual branches or on the top of shoots, shedding up to 30-40% of berries
3.0	Plants are not much weakened, annual growth of up to 25% per bush, flowering up to 50% of at least ½ of the length of the shoot, shedding of berries up to 20%
4.0	The plants have well-leafed bushes, the annual growth is 25-30% per bush, flowering is from 60-70% per bush, the shedding of berries is up to 10-15%
5.0	Healthy plants, good strong annual growth of 40-50% per bush, flowering is along the entire length of the shoot, more than 75%, low berry shedding, individual berries (up to 5%)
